# Cancer Mortality and Incidence of Mesothelioma in a Cohort of Wives of Asbestos Workers in Casale Monferrato, Italy

**DOI:** 10.1289/ehp.10195

**Published:** 2007-07-17

**Authors:** Daniela Ferrante, Marinella Bertolotti, Annalisa Todesco, Dario Mirabelli, Benedetto Terracini, Corrado Magnani

**Affiliations:** 1 Unit of Medical Statistics and Cancer Epidemiology, CPO Piemonte and University of Eastern Piedmont, Novara, Italy; 2 Interdepartmental Centre G. Scansetti, University of Turin for Studies on Asbestos and other Toxic Particulates, Turin, Italy; 3 Unit of Cancer Epidemiology, CPO Piemonte, CeRMS (Centro Ricerca Medicina Sperimentale) and University of Turin, Turin, Italy

**Keywords:** asbestos, domestic exposure, epidemiology, mesothelioma

## Abstract

**Background:**

Family members of asbestos workers are at increased risk of malignant mesothelioma (MM). Although the hazard is established, the magnitude of the risk is uncertain, and it is unclear whether risk is also increased for other cancers. Few cohort studies have been reported.

**Objective:**

The “Eternit” factory of Casale Monferrato (Italy), active from 1907 to 1986, was among the most important Italian plants producing asbestos-cement (AC) goods. In this article we present updated results on mortality and MM incidence in the wives of workers at the factory.

**Methods:**

We studied a cohort of 1,780 women, each married to an AC worker during his employment at the factory but not personally occupationally exposed to asbestos. Cohort membership was defined starting from the marital status of each worker, which was ascertained in 1988 from the Registrar’s Office in the town where workers lived. At the end of follow-up (April 2003), 67% of women were alive, 32.3% dead, and 0.7% lost to follow-up. Duration of exposure was computed from the husband’s period of employment. Latency was the interval from first exposure to the end of follow-up.

**Results:**

The standardized mortality ratio (SMR) for pleural cancer [21 observed vs. 1.2 expected; SMR = 18.00; 95% confidence interval (CI), 11.14–27.52] was significantly increased. Mortality for lung cancer was not increased (12 observed vs. 10.3 expected; SMR = 1.17; 95% CI, 0.60–2.04). Eleven incident cases of pleural MM were observed (standardized incidence ratio = 25.19; 95% CI, 12.57–45.07).

**Conclusions:**

Household exposure, as experienced by these AC workers’ wives, increases risk for pleural MM but not for lung cancer.

Family members of asbestos workers are at increased risk of malignant mesothelioma (MM); although the evidence of association is sufficient [International Agency for Research on Cancer (IARC) 1987], the magnitude of the risk is uncertain. Moreover, the influence of potential determinants of household exposures, such as fiber type and specific activities involving the materials carried home by workers, is poorly understood. Furthermore, it is unclear whether the risk of other asbestos-related malignancies is increased. Evidence of pleural MM in relation to domestic exposure to asbestos has been presented in several case reports and case–control studies (Bourdes et al. 2000; Howel et al. 1997; Magnani et al. 2000, 2001). In contrast, only two cohort studies have been conducted: a cohort study on household contacts of amosite workers (Anderson 1982), and one that we carried out on mortality among the wives of asbestos-cement (AC) workers (Magnani et al. 1993). Our study showed a statistically significant excess in deaths from pleural malignancy, whereas results on lung cancer were not clear-cut because of the limited numbers.

In this article we present the updated results on cause-specific mortality and MM incidence in the cohort of wives of workers employed at the “Eternit” plant in Casale Monferrato (Piedmont, Italy), with > 40 years of follow-up. We also examine the effects of domestic exposure according to duration of exposure and latency.

## Methods

This study was based on a cohort of the wives of AC workers employed at the “Eternit” plant in Casale Monferrato (Italy) on 1 January 1950 or hired between 1950 and 1986. The plant was one of the most important in Italy for the manufacture of AC products, such as high-pressure pipes, plain and corrugated sheets, and chimney tubes. Both chrysotile and crocidolite were used throughout the entire period of activity. Details of the cohort of AC workers and of the factory have been reported previously (Magnani et al. 1996). The factory did not provide laundry facilities, so work clothes were taken home for cleaning.

In Italy, each town has a Registrar’s Office where information on vital and marital status are permanently recorded for each resident. Records of citizens moving to another town can be tracked. In 1988 the Registrar’s Offices of the towns of residence of the AC workers provided us the marital status of each male worker in the AC cohort, together with his wife’s name and birth date. Wives who had died or divorced were included. Information on marital status was available for 2,598 workers of 2,604: 251 were never married, and 2,347 were married, widowed, or divorced (63 had been married twice and 1 three times). The list of wives included 2,410 women (2 women had been married twice, both times to Eternit workers). Furthermore, we excluded 383 women who had worked for any time between 1907 and 1986 at the “Eternit” plant and 9 women for incomplete data (3 with marriage date unknown; 3 with incomplete data about the period of the husband’s employment; and 3 with missing information for follow-up). Eventually, the cohort that was considered for follow-up and estimation of domestic exposure included 2,018 women (Magnani et al. 1993). The cohort membership was not further updated because the factory closed in 1986.

For each woman, the period of domestic exposure was estimated according to the period of time her husband worked in the AC plant. We estimated that exposure began either the date of marriage or the date the husband was hired, whichever was later. We considered the end of exposure to be when the husband ceased employment or the marriage was terminated, whichever came earlier. We excluded 238 women who had no domestic exposure: 224 women married after their husbands had stopped working in the AC plant, and 14 ended their marriages before their husbands were hired. Eventually the exposed cohort included 1,780 women that were considered for the present study. We did not include the unexposed cohort in the present analyses because results were statistically unstable due to the small numbers.

Follow-up was completed on 30 April 2003. Vital status was ascertained through the Registrar’s Offices; the same office provided the cause of death for decedents. For deaths due to pleural and peritoneal malignancy, we examined the clinical records. We coded the underlying cause of death according to the *International Classification of Disease, 9th Revision* (World Health Organization 1975).

Statistical analysis was based on the person-years method. Women in the cohort contributed person-years of observation from the beginning of domestic exposure until their most recent date of observation.

We computed standardized mortality ratios (SMRs; i.e. the ratio of observed to expected deaths using indirect standardization) for the major causes of death. For SMR analyses, the number of deaths expected in the cohort was estimated from age- and sex-specific mortality rates in Piedmont, provided by the National Institute of Statistics - ISTAT (Rome, Italy). Analyses were restricted to person-years and events occurring in 1965–2003, because reference rates were available only for 1970–2002. For the 1965–1969, we applied the rates for 1970–1974. The rates for 2000–2002 were applied to 2003. Therefore, because mortality analyses were limited to 1965–2003, 55 women who had died and 3 who emigrated before 1965 were not considered.

We searched for incident cases of MM in the Mesothelioma Registry of Piedmont (available for 1990–2001). Only incident cases confirmed by histologic or cytologic examination were considered. We computed expected numbers of MM and standardized incidence ratios (SIRs) from the age and sex-specific incidence rates recorded by the Mesothelioma Registry of Piedmont for 1990–2001 (Ivaldi et al. 1999) using indirect standardization. The Mesothelioma Registry of Piedmont also provided information on the occupational history of the cases, as well as on other sources of asbestos exposure.

Both mortality and incidence analyses were limited to person-years and events occurring before 80 years of age in order to avoid the possible misclassification of cause of death or diagnosis in old age. Women contributed person-years and events until 80 years of age and were censored afterward. A short report of the events occurring at ages > 80 years of age is given in the following sections.

Confidence intervals (95% CI) for both SMRs and SIRs were derived, assuming a Poisson distribution for the number of observed events. Statistical analyses were carried out using OCMAP-PLUS, release 3.10 (University of Pittsburgh, Pittsburgh, PA, USA) (Marsh et al. 2002) and SAS, release 8.2 (SAS Institute Inc., Cary, NC, USA).

## Results

Table 1 presents the composition of the cohort. At the end of follow-up, 67% of the women were alive, 32.3% had died (with cause of death known for 97.1%), and 0.7% were lost to follow-up or had moved abroad. During 1965–2003 the cohort contributed 51,873 person-years of follow-up in total.

Mortality from all causes and from all malignant neoplasms was near expectations (Table 2). We observed a statistically significant increase in mortality for malignant neoplasms of the respiratory system (SMR = 2.69; *p* < 0.01) and for malignant neoplasms of the pleura (SMR = 18.00; *p* < 0.01). Other diseases of *a priori* interest, such as peritoneal neoplasm (3 observed vs. 1.2 expected), lung cancer (12 observed vs. 10.3 expected), cancer of the ovary (11 observed vs. 7.7 expected), and nonmalignant respiratory diseases (14 observed vs. 16.2 expected), did not show a statistically significant increase in mortality. A statistically significant increase in the number of deaths was observed for psychiatric disorders and for poorly defined causes, whereas mortality for diabetes and external causes was significantly decreased. The diagnosis of pleural malignancy as a cause of death was supported by histologic examination in 13 cases and by cytologic examination in 2; 1 case was diagnosed by radiologic examination. No further information was available for the remaining 5 cases.

Mortality for pleural malignancy according to duration of domestic exposure and latency is shown in Table 3. In all categories of duration of exposure the SMR was significantly increased, with higher SMRs for the longer duration categories. With regard to latency, a statistically significant increase in SMRs was observed at least 30 years after first exposure. The shortest observed latency and duration of exposure were 11 years and 5 years, respectively. Table 3 also presents the joint analysis of latency and duration of exposure: Results showed an increase in SMRs in relation to both variables, but the trend was irregular because of small numbers.

Table 4 presents the incidence of MM overall and by latency and duration of exposure. Overall 11 cases were observed against 0.44 expected (SIR = 25.19; 95% CI, 12.57–45.07) during 1990–2001. SIRs increased with duration of exposure. SIRs showed a statistically significant increase after 30 years of latency; only one case occurred before 30 years of latency. The joint analysis by latency and exposure did not show clear results, but the number of cases was very small.

Analyses of mortality and MM incidence were also carried out by calendar period of first exposure. No statistically significant differences were observed among periods, although there was a suggestion for lower risks in the more recent periods. SMRs for pleural malignancy were as follows: 16.8 (95% CI, 6.2–36.6) for women starting exposure before 1950; 20.4 (95% CI, 11.1–34.2) for exposure starting in 1950–1964, and 7.7 (95% CI, 0.04–57.2) for exposure starting after 1964. Similar results were observed for MM incidence: SIRs were 42.0 (95% CI, 5.1–152) for women starting exposure before 1950; 25.9 (95% CI, 11.2–51.1) for women starting exposure in 1950–1964, and 12.5 (95% CI, 0.06–92.9) for women starting exposure after 1964.

According to the information in the MM registry, four of the women affected by MM also had fathers who were occupationally exposed at the Eternit plant, and another had herself worked as a goldsmith for 12 years, with possible exposure to asbestos.

## Discussion

Many studies of different epidemiologic design have examined the effects of asbestos exposure at work (Albin et al. 1999); more limited evidence is available on whether the occurrence of cancer increases after domestic and residential exposure and on the magnitude of increased risks (Bourdes et al. 2000). As far as we know, only two cohort studies have been published on cancer in family members of asbestos workers: the first by Anderson (1982) in relation to amosite workers in Paterson, New Jersey (USA), and the second by us (Magnani et al. 1993) in relation to AC workers in Casale Monferrato (Italy). Among female household contacts of asbestos workers, Anderson (1982) found 8 deaths from respiratory cancer versus 4.7 expected after 20 years from the onset of exposure. Two of the deaths were from mesothelioma.

The main result of the present study is the statistically significant increase in both pleural cancer mortality and pleural MM incidence among women who have been exposed to asbestos at home as wives of AC workers. The SMR for pleural neoplasm was higher (18.0) than observed in our previous study (8.0; Magnani et al. 1993), which is likely to reflect the longer latency covered by the longer follow-up. The methods and the cohort were the same. Results from the incidence study showed a higher risk (SIR = 25.19; 95% CI, 12.57–45.07) than the mortality study (SMR = 18.00; 95% CI, 11.14–27.52), which is probably because of misclassification of causes of death. However, the two results are not exactly comparable because the analysis of incidence covered the period 1990–2001, whereas the mortality analysis covered the period 1965–2002 (i.e., including a larger contribution of person-years in younger ages and in shorter exposure and latency classes).

Pleural MM was the only cancer for which we observed a statistically significant increase in risk. Mortality from lung cancer was increased only modestly and not to the point of statistical significance. In respect to lung cancer, the present study has 53% power for observing a relative risk (RR) of 1.65 (i.e., the finding in our previous study) and 82% power for observing an RR of 2 as statistically significant with α = 0.05 using a two-tailed statistical test (Breslow and Day 1989). The category of “psychiatric disorders” also showed a statistically significant excess mortality, whereas mortality from diabetes and from violence was significantly reduced; none of these causes is thought to be related to asbestos exposure.

In all categories of exposure duration, SMRs for pleural neoplasm were significantly increased and we observed an increasing trend with longer duration of exposure. Regarding latency, the SMR reached statistical significance after 30 years from first exposure. We observed a similar pattern in relation to MM incidence. These results are consistent with the findings from cohort studies of occupationally exposed subjects.

The cohort in the present study is unique because of its size and the duration of follow-up as well as its link to an occupational cohort study. The women in the cohort and the follow-up were entirely based on official records: employees’ rosters and town office archives. Recall or selection biases are therefore unlikely. We took great care in excluding from the cohort the women with direct occupational exposure at the Eternit plant. The search was based on maiden name, which in Italy continues to be used in official records after marriage. However, to avoid any possible mistake, we also repeated the search using husband’s name; no further linkages were found. There have been no other asbestos-using plants in the area, so the women had few opportunities for occupational exposure to asbestos from other industrial sources. Among the 11 incident cases of mesothelioma, we detected only one woman with occupational exposure—she had worked as a goldsmith.

We had no quantitative information on the extent of asbestos exposure in the homes in the Casale Monferrato area. Little information is available in the literature, and its relevance to Casale Monferrato is questionable. For instance, from an extensive survey in the chrysotile mining area of Quebec, Camus et al. (1998) estimated an average cumulative concentration of 7.8 fiber-years/mL for household exposure in women. Data reviewed by Bourdes et al. (2000) indicated a very large variation in indoor exposure levels, with the higher concentration measured in the homes of South-African miners (range, 2–11 fibers/L).

We compared mortality in our cohort with regional rates, which were more appropriate than national rates because of the wide regional differences in respiratory cancer mortality in Italy (Cislaghi et al. 1986). Regarding pleural cancer, the regional population is also more appropriate than the national one because mortality from pleural cancer is different (higher) in Piedmont, and in general varies widely among Italian regions (Mastrantonio et al. 2002). Piedmont has 4.5 million inhabitants; therefore, death rates will not have been importantly affected by mortality in the cohort under investigation.

For the analysis of MM incidence, both the incident cases and the reference rates were provided by the regional MM registry (Ivaldi et al. 1999). The incidence rate for pleural MM in women in Piedmont during 1999–2001 was 1.2/100,000 person-years, based on an average of 38 cases/year. The contribution of the present cohort to the regional rate was approximately one incident case per year and therefore not large enough to influence the reference rate that was used.

Analyses were limited to women < 80 years of age in order to counter possible biases from the misclassification of causes of death and the loss of incident MM cases because of inadequate diagnosis in the elderly. Although occupational cohort studies are not usually age-censored, age censoring has been used by other authors dealing with MM incidence in the general population (Järvholm et al. 1999). The possible bias in estimating the relative risk including older age classes is unlikely to be large because the same inaccuracy would apply to both observed and expected numbers, but the attributable number of cases (i.e., observed –expected) would be underestimated. In respect to mortality in the age class > 80 years of age, we observed 1 death from pulmonary cancer and 3 deaths from pleural cancer, while 1.64 and 0.2 were expected, respectively. In respect to incidence, a loss of incident MM cases at older ages seems likely. The Piedmont Mesothelioma Registry observed a reduction in incidence rates for histologically confirmed MM in older age classes, probably because diagnostic investigation is less invasive in the elderly. Only one incident case of MM was observed in the present study after 80 years of age.

Many subjects in the present study have been living in the town of Casale Monferrato, where environmental exposure occurred and may have contributed to the risk observed in the present study (Magnani et al. 2001). However, the cohort study design of the present study precluded us from including the full residential history among the variables that were considered.

The risk estimates from the present study are consistent with those of other studies of cohabitants with asbestos workers. Spirtas et al. (1994) observed a 13-fold increase in risk for men and 3-fold increase for women for pleural and peritoneal mesothelioma together (analyses ignored occupational exposure). In a case–control study in northern England, Howel et al. (1997) estimated an odds ratio (OR) of 5.8 for paraoccupational exposure (not better specified). In our previous international study (Magnani et al. 2000), the OR was 7.8 (95% CI, 1.7–36.2) for domestic exposure from cohabitation with an asbestos worker and handling of his/her work clothes. In a case–control study in the area, we measured an RR of 3.1 for spouses (both sexes) of AC workers (SMR = 792; 95% CI, 216–2,029) (Magnani et al. 2001). We observed higher risk estimates in the present study because we focused on wives only (i.e., the persons who were more heavily exposed in the household because of dust from handling and cleaning work clothes). Because of the rarity of disease, case series also provide useful information on the risk of MM in wives of asbestos workers. Several such studies have been published since 1990: Roggli and Longo (1991) reported on six women with respiratory cancers among household contacts of asbestos workers; three were affected by pleural MM and three by lung cancer with markers of asbestos exposure. Dodoli et al. (1992) reviewed death certificates from 1975–1988 in Livorno and La Spezia (Italy). They found 10 cases of pleural MM in a total of 56 women that they attributed to domestic exposure from cleaning the work clothes of asbestos-exposed relatives; 8 of the cases were wives of exposed workers. Bianchi et al. (2004), in a study on MM in a ship-building district in northeastern Italy, reported five patients who had been exposed at home while cleaning their relatives’ working clothes. A study carried out in the United States reported 32 MM cases with domestic asbestos exposure from relatives employed in asbestos-associated industries (Miller 2005). A meta-analysis on MM and environmental exposure to asbestos included one ecologic study, one cohort study, and three case–control studies on household exposure. Bourdes et al. (2000) estimated an overall RR of 8.1 (95% CI, 5.3–12.0), with the RR in the individual studies ranging between 4.0 and 23.7.

In conclusion, the present study confirms the increased risk of MM from home exposure among women married to asbestos workers. The increase of risk was limited to pleural MM and was not observed for other diseases associated with occupational asbestos exposure, such as lung cancer.

## Figures and Tables

**Table 1:** Characteristics of the cohort of wives of AC workers in Casale Monferrato, Italy.

Characteristic	No.
Total women	1,780
Women excluded as lost or dead before 1965	58
Women with follow-up after 1 January 1965	1,722
Vital status (limited to subjects with follow-up after 1 January 1965)	
Alive on 30 April 2003	1,106
Dead after 1965	606
Cause of death unknown	16
Moved abroad after 1965	7
Lost to follow-up	3
Birth year (mean ± SD)	1926 ± 14.9
Year of marriage (mean ± SD)	1950 ± 14.5
Duration of exposure (mean ± SD)	15.0 ± 9.5
Person-years of observation (from 1965), by age class	
15–19	43
20–24	537
25–29	1,465
30–34	2,641
35–39	4,079
40–44	5,288
45–49	5,863
50–54	6,240
55–59	6,393
60–64	6,075
65–69	5,124
70–74	3,759
75–79	2,304
≥80	2,062
Total	51,873

**Table 2:** Cause-specific mortality before 80 years of age of the cohort of wives of AC workers in Casale Monferrato, Italy, during 1965–2003.

	Women with domestic exposure		
Cause of death	Observed	Expected	SMR (95% CI)
All causes	396	389.3	1.02 (0.92–1.12)
Malignant neoplasm	146	136.1	1.07 (0.91–1.26)
Digestive organs and peritoneum	49	47.5	1.03 (0.76–1.36)
Intestine and rectum	21	16.0	1.31 (0.81–2.00)
Rectum	10	5.0	2.00 (0.96–3.69)
Peritoneum	3	1.2	2.51 (0.52–7.35)
Respiratory organs	33	12.3	2.69 (1.85–3.77)**
Lung	12	10.3	1.17 (0.60–2.04)
Pleura	21	1.2	18.00 (11.14–27.52)**
Breast	20	29.1	0.69 (0.42–1.06)
Uterus	5	10.9	0.46 (0.15–1.07)
Ovary	11	7.7	1.42 (0.71–2.54)
Nervous system	6	3.4	1.77 (0.65–3.86)
Bladder	2	1.8	1.10 (0.13–3.99)
Unspecified	4	3.1	1.29 (0.35–3.31)
Diabetes	4	13.6	0.29 (0.08–0.75)*
Psychiatric disorders	8	2.1	3.83 (1.65–7.54)**
Cardiovascular diseases	145	152.9	0.95 (0.80–1.12)
Ischemic cardiopathy	28	40.1	0.70 (0.46–1.01)
Respiratory diseases	14	16.2	0.86 (0.47–1.45)
Digestive diseases	25	23.9	1.05 (0.68–1.55)
External causes	8	16.9	0.47 (0.20–0.93)*
Poorly defined	14	2.9	4.86 (2.66–8.16)**

* *p* < 0.05.

** *p* < 0.01.

**Table 3:** Mortality from pleural neoplasms in the cohort of wives of AC workers in Casale Monferrato, Italy, during 1965–2003, by latency and duration of exposure.

	Duration of exposure (years)											
	0–9			10–19			≥ 20			Total		
Latency (years)	Obs	Exp	SMR (95% CI)	Obs	Exp	SMR (95% CI)	Obs	Exp	SMR (95% CI)	Obs	Exp	SMR (95% CI)
< 20	0	0.10	—	1	0.05	18.96 (0.47–105.63)				1	0.15	6.74 (0.17–37.56)
20–29	1	0.11	9.47 (0.24–52.75)	1	0.10	10.43 (0.26–58.12)	0	0.06	—	2	0.27	7.54 (0.91–27.23)
30–39	2	0.11	18.47 (2.23–66.72)*	5	0.15	34.41 (11.17–80.30)**	4	0.17	23.75 (6.47–60.82)**	11	0.42	26.07 (13.01–46.64)**
≥ 40	0	0.04	—	0	0.07	—	7	0.22	31.96 (12.85–65.84)**	7	0.33	21.17 (8.51–43.61)**
Total	3	0.35	8.51 (1.76–24.88)*	7	0.36	19.30 (7.76–39.76)**	11	0.45	24.37 (12.17–43.61)**	21	1.17	18.00 (11.14–27.52)**

Abbreviations: Exp, expected; Obs, observed. Subjects were censored at 80 years of age.

* *p* < 0.05.

** *p* < 0.01.

**Table 4:** Incidence of MM of the pleura diagnosed histologically or cytologically in the cohort study of wives of AC workers in Casale Monferrato, Italy, during 1990–2001, by latency and duration of exposure.

	Duration of exposure (years)								
	0–19			≥ 20			Total		
Latency (years)	Obs	Exp	SIR (95% CI)	Obs	Exp	SIR (95% CI)	Obs	Exp	SIR (95% CI)
< 30	1	0.08	12.26 (0.31–68.34)	0	0.01	—	1	0.09	11.28 (0.28–62.88)
30–39	5	0.14	36.96 (12.0–86.25)**	1	0.07	14.14 (0.35–78.79)	6	0.21	29.13 (10.69–63.39)**
≥ 40	0	0.06	—	4	0.08	50.59 (13.78–129.53)**	4	0.14	28.15 (7.67–72.07)**
Total	6	0.28	21.44 (7.87–46.66)**	5	0.16	31.87 (10.35–74.38)**	11	0.44	25.19 (12.57–45.07)**

Abbreviations: Exp, expected; Obs, observed. Subjects were censored at 80 years of age.

** *p* < 0.01.
